# Mind Cure

**Published:** 1887-11

**Authors:** 


### MIND CURE.

"We affirm that Mind Cure in and of itself means simply that the the mind must cure whatever is wrong both in mind and body, and the universal specific is mental and not physical. 'Who shall minister to a mind diseased?' is the question continually asked by sufferers. How long will physicians continue to treat ailments which are purely mental as though they were bodily? is a question that comes up in all our popular literature. We need greater sagacity and a much wider sweep of intelligence to reach the mind than merely to reach the body: the endeavor to tinker up the flesh while the mind is ill at ease is of no use whatever. The endeavor to cure people from dyspepsia when it is not their food that disagrees with their stomach, which is not out of order except as an after consequence, for their ailment from mental unrest, from grief, disappointment and unhappiness, from something that weighs upon the mind, a heavy load upon the heart, a sting of conscience rebuking them for an error, is all in vain when you rely on pills, powders and balsam. If you could get at the reason why people suffer from dyspepsia, if you could get at the reason why good food makes them sick, or remains undigested, if you could get at the reason why they are unhappy and unable to obtain relief, you would then be able by dealing with and removing the cause of the unhappiness to heal them. If you could not remove the thorn from the mind which afterwards produced the semblance of a thorn rankling in the flesh, you wonld at least be able to do what a spiritual teacher was able to accomplish in his own case—help them to receive from heaven grace sufficient to bear it." *Colville.*

